# Exploring the Relationship Between Corporate Social Responsibility, Trust, Corporate Reputation, and Brand Equity

**DOI:** 10.3389/fpsyg.2021.766422

**Published:** 2021-11-10

**Authors:** Yan Zhao, Manzir Abbas, Madeeha Samma, Tarik Ozkut, Mubbasher Munir, Samma Faiz Rasool

**Affiliations:** ^1^School of Management, Shanghai University, Shanghai, China; ^2^Department of Business Administration, Institute of Social Sciences, Istanbul Okan University, Istanbul, Turkey; ^3^Department of Economics and Statistics, Dr. Hasan Murad School of Management, University of Management and Technology, Lahore, Pakistan; ^4^School of Management, Zhejiang University of Technology, Hangzhou, China

**Keywords:** corporate social responsibility (C.S.R.), trust, corporate reputation behavior, brand equity, social perception

## Abstract

The purpose of this study is to investigate the relationship between corporate social responsibility (CSR), corporate reputation (CR), and brand equity (BE). Building on the resource-based theory of the firm, this study proposes a theoretical framework. In this framework, CSR is theorized to strengthen CR and brand equity, directly and indirectly, through consumer trust. We used a questionnaire survey approach. In the questionnaire, 17 items were used with a 5-point Likert-Scale (1 stands for “strongly disagree,” and 5 stands for “strongly agree”). Data were collected from the consumers of the banking sector in the vicinity of Lahore, Pakistan. To estimate the proposed relationships in the conceptual model, we use structural equation modeling (SEM) through Smart PLS 3.2. The outcomes of this study confirm that CSR significantly impacts CR and brand equity. It is also demonstrated that trust mediates positively and significantly in the relationship between CSR, CR, and BE. Results of the present study have several implications for the senior management, marketing expert, administrators, and policymakers. This study expresses how CSR boosts BE and CR. Moreover, this study also indicates that trust is an important factor that enhances BE and CR.

## Introduction

Corporate social responsibility (CSR) is attracting a lot of interest and attention in the competitive world market (Abbas et al., 2019). The importance of CSR may also be revealed in the fact that firms are spending millions on CSR (Luo and Bhattacharya, 2006). However, CSR is an active contributor to building a good reputation and brand equity (BE) in the Pakistani banking sector, particularly in public sector banks (PSBs). Abbas et al. (2019), argue that CSR is a form of international private business self-regulation which aims to contribute to societal goals of a philanthropic, activist, or charitable nature by engaging in or supporting volunteering or ethically oriented practices. According to Smith (2003), the success of an organization depends on the strategies that organizations develop to increase their sales through CSR (Zhou et al., 2021). Therefore, organizations build moral capital or assets in intangible forms such as BE and corporate reputation (CR) by investing more in CSR activities. The moral capital of the company acts as an insurance policy, protecting it from negative stakeholder assessments. Companies can also increase their BE by participating in CSR (Fatma et al., 2015).

According to Lai et al. (2010), BE and CR are considered critical intangible assets to the success of a company in the financial services sector. It is also suggested that a key variable in the consumer-organization association is trust. It has been understood that trust is a factor while considering the prospects of a buyer about behavior regarding CSR perspective (Vlachos et al., 2009). When customers believe an organization is trustworthy and behaves in a socially responsible manner, the evaluation and assessment of a company may be positively affected (Edinger-Schons et al., 2019). In the Pakistani context, the banking sector has been growing and strengthened at a sustained pace with growth in its deposits, advances, and overall profitability. As per the quarterly performance review of the SBP banking sector report from January to June 2018, asset growth in the banking sector is 4.7%, and it stood at Rs. 19,197.1 billion, whereas deposits stood at $13,755 billion (Zia et al., 2021).

The lack of attention paid with respect to contributing to society and the well-being of the community in which they operate has been noted. Furthermore, PSBs generally serve the federal government as well as various provincial governments. As a result, the government becomes its main customer, and they amuse and attract a regular customer base in order to compete in a competitive market (Arshed et al., 2021). As a result, PSBs have strong roots and are well-entrenched in society, and they are seen as a key participant in achieving financial inclusion, financial literacy, and offering commercial services to the general people. However, they are also unaware of their role as CSR participants and the benefits of becoming a good corporate citizen by participating in CSR.

One possible reason is that PSBs are not cognizant that investing in CSR can greatly help build their reputation and brand equity, further attracting customers and their loyalty, thereby increasing their overall profitability. The PSB management and board of directors seem unaware and ignorant about their critical role as CSR participants and good corporate citizens, which can substantially help build a reputation and BE for their organization. Unfortunately, no such study on CSR activities for PSBs in Pakistan has been found that endorses the concept and theory of CSR initiatives that can contribute toward building reputation and brand equity. The results of such a study can significantly help to attract the attention of management and the board of directors of PSB in deciding their future course of action to CSR activities. Based on the above literature insights about CRS, trust, BE, and CR, this study proposes the following questions:

**RQ1.** Does CSR influence BE and CR?

**RQ2.** How does trust intervene between BE, CR, and CSR?

This study is organized as follows: section “Hypotheses Framing and Conceptual Framework” describes the hypotheses framing and conceptual model. Section “Research Methods” presents the research methods. Section “Results” is about the data analysis and results, whereas section “Discussions” highlights the discussion of this study. Finally, section “Conclusion” is about the conclusion, limitation, and future research directions.

## Literature Review

### Corporate Social Responsibility

Integration of the interests and business activities of societies needs to be aligned for all organizations operating in society to be beneficial for both organizations and society. According to Crane et al. (2008), “corporate social responsibility should be considering as a strategic investment form that viewed in establishing or maintaining the corporate reputation,” and this suggests that direct and indirect execution of CSR are viewed *via* resource-based view (RBV) of a company when these types of leads executions and exercises impact the advantages of the firms. The manageable upper hand can be collected from these immaterial resources on the off chance that they are uncommon, important, and supreme (Shin and Thai, 2015). To comprehend why firms participate in socially mindful exercises, RBV fills in as a valuable apparatus (Branco and Rodrigues, 2006). Customers separate an item from two points of view, for example, vertical separation and even separation. Vertical separation implies the inclinations of a customer for buying items from socially dependable firms, in contrast with others. Vertical separation fortifies CR, enhances BE, and enables the organization to charge additional prices (Castelo Branco and Lima Rodrigues, 2006). Even separation implies that the inclination of shoppers to buy certain items depends on their taste. This study does not enable the organization to charge a premium cost as it has not increased the value of CR also (Ghaderi et al., 2019).

### Corporate Reputation

Newburry et al. (2019) define CR as a collective perception of the past activities and beliefs of the firm regarding its future activities. Moreover, Gotsi and Wilson (2001) demonstrate that CR is the future marketing plan that will affect the internal and external stockholders of the organization. Jeffrey et al. (2019) express that CR is a reputation that brings trust and loyalty between consumers and vendors. Furthermore, a good business reputation brings a novel employee recruitment process, employee development, and employee retention. CR refers to the degree and level of a firm being considered and placed in great regard for the perceptions of its partners (Newburry et al., 2019). It can also be considered to summarize all perceptions of the stakeholders toward a firm regarding how it will fulfill or exceed the anticipations (Rettab and Mellahi, 2019; Hameed et al., 2021). Also, the reputation of a firm is governed by the indicators of the marketplace regarding its behavior, as understood by stakeholders (Jeffrey et al., 2019).

### Brand Equity

Brand equity is a slogan that is used in the marketing industry. The BE shows the value of a well-known brand label. So, based on this idea, organizations develop well-known brand names that increase the revenue of the organizations. Therefore, consumers believe that a well-known product is better than those products that do not have well-known names in the market. Ahmad I. et al. (2019) demonstrate that BE can be referred to as “the additional benefit or maximum worth that increased a product due to its brand name.” Moreover, Hoang et al. (2019) note that a financial approach has been utilized to examine BE. The financial approach indicators include share value fluctuations and accounting base value, where consumer-centric indicators include perpetual (Chang, 2017; Raji et al., 2019).

## Hypotheses Framing and Conceptual Framework

### Corporate Reputation and Corporate Social Responsibility

Corporate social responsibility is a self-regulatory business model that enables a firm to be socially accountable to the organization, stakeholders, and the general public (Farid et al., 2019). CSR allows a company to be aware of its impact on all elements of society, including economic, social, and environmental issues, and being a socially responsible firm can help the image and brand of a company. As a result, CSR allows employees to use the resources of a company to accomplish well (Kim et al., 2017). CR is considered an impalpable, value resource by any firm. It serves as a crucial factor in determining the competitive benefit, particularly in a product where diversity is negligible in the consumer (Arikan et al., 2016; Baudot et al., 2019).

According to Benitez et al. (2017), consumers assess and appraise new services or products launched based on their existing image in the market. Moreover, an excellent CR provides a shield against adverse customer perspectives because CR results from its business activities. In contrast, CSR initiatives are considered the best profitable method to construct a good reputation and perception in consumers and stakeholders (Lee et al., 2017; Lee, 2019). Thus, engaging in publicly accountable activities and contributing to the well-being of a society enhances the image and reputation of a company (Gulzar et al., 2018). As per Melero-Polo and López-Pérez (2017), the consumer perspective of CSR initiatives impacts affirmatively in building CR. Forcadell and Aracil (2017) endorse that suitable social activity of firms leads to increased CR. Previous studies also indicate that CSR positively correlates with a CR (Brammer and Pavelin, 2016; Gardberg et al., 2019; Khan et al., 2020a). Based on the above discussion, we proposed our first hypothesis:

**H1.** CSR positively influences CR

### Corporate Social Responsibility and Brand Equity

The concept of BE has been argued in both accounting and marketing literature, and it has underlined the necessity of a long-term perspective in brand management (Khan et al., 2020b). Although businesses have made substantial efforts to manage brands strategically, there is still a lack of consistent terminology and philosophy inside and between disciplines, which can obstruct communication. Accountants and marketers have varied definitions of brand equity, with the idea being described both in terms of the customer-brand connection and as something that accrues to the brand owner. Organizations build BE for their products by making them distinctive, easily recognizable, and high-quality and reliable (Yang and Basile, 2019; Yang et al., 2020).

Prior studies highlighted that BE has a positive relationship with CSR (Abdolvand and Charsetad, 2013; Lin and Chung, 2019; Yang and Basile, 2019). BE was originated from the strong interaction between brands and consumers (Martínez and Nishiyama, 2019). The more consumer expectations are met or exceeded, the more the BE (Liu and Lu, 2019). Similarly, the ethical behavior of a firm will lead to its reputation and a crucial indicator of brand appraisal (Beckmann, 2007). Thus, the study assumes that the customer perspective of CSR initiatives may positively impact brand equity. So, we proposed our second hypothesis:

**H2.** CSR positively influences BE

### Corporate Social Responsibility and Trust

Business ties are held together by a social glue called trust. Business partners that trust each other spend less time and energy defending themselves from being exploited, and both sides achieve greater financial results in negotiations (Farid et al., 2020). When your employees know that you trust them and that they can express themselves clearly, trust becomes vital in business. They will almost certainly be more driven to be productive and work to their best potential (Park et al., 2014).

Looking into previous literature, we have provided concepts to understand the relationship between CSR and trust (Pivato et al., 2008; Vlachos et al., 2009; Park et al., 2014). So, the results of these studies confirm that CSR has a positive and significant relationship with trust. Moreover, the image of an organization and its norms help establish trust in the company that can be derived from its socially responsible initiatives (Azmat and Ha, 2013). Organizations that are perceived as publicly answerable by consumers are inclined to have high trust levels in the eyes of the consumers (Cheung and Pok, 2019; Kim, 2019). Hence, our third hypothesis is presented below:

**H3.** CSR positively influences trust.

### The Mediating Effects of the Trust

**FIGURE 1:**
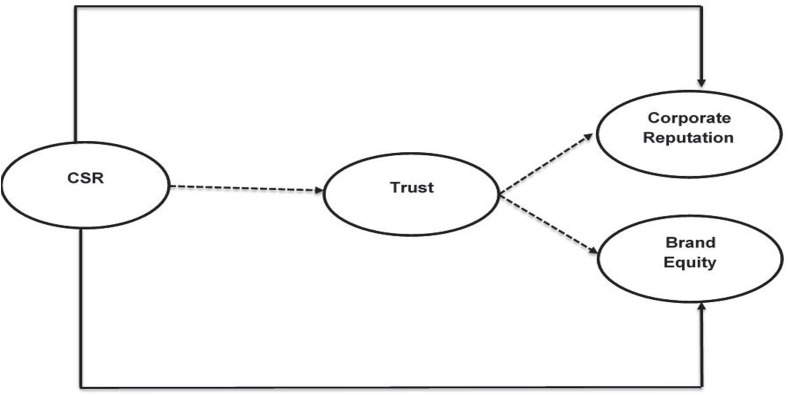
Conceptual model. Solid lines show the direct relationship, and dashed lines show the indirect relationship.

According to the study of Iglesias et al. (2018), the benevolence base trust includes customer perception as either a firm honestly serious or concerned about the well-being and welfare of society. Similarly, another famous author, Arikan et al. (2016) indicated by the social trade hypothesis that a client trust in the direction of the firm image improves the social integration of the client association to build client responsibility toward the brand (Nguyen and Pham, 2018; Kim, 2019; Zhao et al., 2019). The consumer does an overall assessment and evaluation of the image of the firm, which is drawn on their perception and information about the firm (Joo et al., 2017), and trust is the forerunner to CR. Many previous studies also confirm in their results that trust is the mediator between CRS and CR and brand equity. Therefore, the literature suggests that trust is positively and significantly mediates between the CRS, CR, and brand equity. Figure 1 is presenting the conceptual model of this study. Thus, this research makes the following hypotheses:

**H4.** Trust mediates CSR and BE
**H5.** Trust mediates between CSR and CR

## Research Methods

### Research Approach

In this study, we used a questionnaire survey analysis approach. We used this approach because it is common and has a broad sample of the given population that can be contacted at a relatively low cost (Roby et al., 2003; Heeringa et al., 2017; Wang et al., 2021). Hennessy and Patterson (2011) suggested that for the survey analysis, first, we should develop the research questionnaire (Rasool et al., 2019a,b). So, in this study, first, we designed the questionnaire to collect data.

### Questionnaire Designing

This study aims to explore the influence of CSR, directly and indirectly, on CR and brand equity, using trust as a mediating variable. In this questionnaire, 17 items were used with a 5-point Likert-Scale (1 stands for “strongly disagree,” and five stands for “strongly agree”). The questionnaire is consists of five sections. The first section includes the demographic information of the respondents. The second section took insight of the customers about CSR activities. The third part of the questionnaire measured the respondent’s level of trust in the banking sector. The fourth part of the questionnaire explained the CR -related information, and the last and fifth sections encompassed acclaimed variables that measure BE dimensions. Before data collection, the authors also conducted a pilot study to check the reliability and validity of the questionnaire. The pilot study respondents suggested some modifications to the questionnaire. Therefore, the questionnaire was revised as per the recommended feedback from the pilot study respondents. So the revised questionnaire was distributed for data collection.

### Variables Measurements

The items of CSR are adopted from López-González et al. (2019). All items of CSR were measured with 5-points Likert-Scale (1 “strongly disagree” and 5 “strongly agree”). The alpha of CSR was 0.920. The results indicate that the factor loading of each item is more than the standard value (0.70). So in this study, measures were considered adequate. The factor loading of each item is mentioned in Table 1.

**TABLE 1:** Construct reliability and validity.

	**Alpha**	**rho_A**	**CR**	**AVE**
Brand equity	0.917	0.920	0.941	0.801
CSR	0.935	0.938	0.959	0.885
Corporate reputation	0.920	0.922	0.940	0.758
Trust	0.939	0.942	0.954	0.805

The items of CR were adopted by Suki and Suki (2019). All items related to CR were measured with a 5-point Likert-Scale (1 “strongly disagree” and 5 “strongly agree”). The alpha of CR was 0.920. The results indicate that the factor loading of each item is more than the standard value (0.70). So in this study, measures were considered adequate. The factor loading of each item is mentioned in Table 1.

The items of BE are adopted from previous studies Çifci et al. (2016). For the measurement of brand equity, we apply the 5-point Likert-Scale (1“strongly disagree” and 5 “strongly agree”). The alpha of BE was 0.917, which is appropriate. The results indicating that the factor loading of each item is more than the standard value (0.70). So, the alpha values met the threshold criteria. The factor loading of each item is mentioned in Table 1.

We use to trust the items developed by Tzempelikos and Gounaris (2017). All items were measured on a 5-points Likert-Scale (1“strongly disagree” and 5 “strongly agree”). The alpha of trust was 0.939, which is acceptable. The results indicate that the factor loading of each item is more than the standard value (0.70). So, the alpha values met the threshold criteria. The factor loading of each item is mentioned in Table 1.

### Data Collection

The data was collected using self-administered questionnaires from the consumers of banking sectors living in Lahore city, the capital of Punjab province of Pakistan. The banking sector consumers for this research refer to those people who are aged above 18 years and have an account in any bank during the survey. Data were collected between 2018 and 2019. This survey was conducted during the working hours of the banks. A non-probabilistic sampling technique with a combination of convenience sampling techniques was used for this research to measure demographic variables like respondent gender, qualification, age, and income. Above all, variables ensure that the sample signifies the sociodemographic features of the present population. At present, for this study, initially, a total of 550 survey instruments were circulated, and from these, 342 questionnaires were received and 23 castoffs due to incomplete information that leaves us with 319 responses. Also, 10 unusable ware responses were identified, leaving 309 responses for the final analysis of this research. The majority of respondents in this research were men, around 57.09%, and women 46.91% aged between 20 and 60 years. Most of the audience holds a bachelor’s degree 950 (16%) and belongs to middle-income families earning $200–690 monthly (43.89%). For more detailed analysis, all variables were inspected for data entry errors, missing values, and the fit between distributions using the software SmartPLS.

## Results

The relationships drawn in the conceptual model were examined using the SEM method. The rationale for choosing SEM over the covariance-based SEM approach is that it is less vulnerable to sample size.

The degree to which all of the multiple elements of the model are used to test its convergent validity (Kura, 2017), shown in Table 2. For this, the threshold value should be >0.6 (Hair et al., 2016). Since all of our values met the threshold requirement, each data collection indicator is valid.

**TABLE 2:** Factor loading.

	**Brand equity**	**CSR**	**Corporate reputation**	**Trust**
BE1	0.922			
BE2	0.886			
BE3	0.927			
BE4	0.842			
CR1			0.873	
CR2			0.887	
CR3			0.841	
CR4			0.902	
CR5			0.849	
CSR1		0.961		
CSR2		0.953		
CSR3		0.908		
TR1				0.883
TR2				0.918
TR3				0.907
TR4				0.890
TR5				0.887

The degrees that display actuality or affirm convergent validity are referred to as average variance extracted (AVE) (Ahmad S. et al., 2019). The AVE value should be larger than 0.5, according to Fornell and Larcker (1981). The reliability of structures has been demonstrated using composite reliability and Cronbach’s alpha value. The stability of structures has been measured using composite reliability and Cronbach’s alpha value. According to Hair Jr. and Sarstedt, it should have a value larger than 0.7; many of the variables in our sample have values that are greater than or equal to the threshold value.

Discriminant validity demonstrates that objects and their constructs have distinct meanings (Hair et al., 2016). Its value is higher than 0.6 in Table 3, which represents valid results, while negative results indicate the reverse.

**TABLE 3:** Discriminant validity of constructs.

**Sr. no**	**Variables**	**1**	**2**	**3**	**4**
1	Brand equity	0.895			
2	CSR	0.777	0.941		
3	Corporate reputation	0.863	0.884	0.871	
4	Trust	0.817	0.843	0.887	0.897

Cross loading is a technique for demonstrating that the loading value of the indicator is highest with one construct and lowest with another (Hair et al., 2017). Table 4 reveals that the values of indicators are adequate for their construct but not for others.

**TABLE 4:** Cross loading.

	**Brand equity**	**CSR**	**Corporate reputation**	**Trust**
BE1	0.922	0.765	0.803	0.771
BE2	0.886	0.635	0.739	0.637
BE3	0.927	0.663	0.753	0.760
BE4	0.842	0.706	0.789	0.743
CR1	0.679	0.812	0.873	0.746
CR2	0.795	0.828	0.887	0.804
CR3	0.761	0.672	0.841	0.719
CR4	0.764	0.766	0.902	0.812
CR5	0.761	0.760	0.849	0.778
CSR1	0.769	0.961	0.881	0.832
CSR2	0.712	0.953	0.835	0.787
CSR3	0.711	0.908	0.776	0.757
TR1	0.748	0.826	0.810	0.883
TR2	0.772	0.825	0.819	0.918
TR3	0.759	0.794	0.819	0.907
TR4	0.710	0.651	0.746	0.890
TR5	0.668	0.661	0.780	0.887

The standardized root mean square residual (SRMR), which is based on converting both the sample covariance matrix and the predicted covariance matrix into correlation matrices, is a measure of the mean absolute value of the covariance residuals. The difference between the observed correlation and the model indicated correlation matrix is defined as the SRMR. As a result, it is possible to use the average size of the differences between observed and anticipated correlations as an absolute measure of (model) fit. A good fit is defined as a value <0.10 or 0.08 (Hu and Bentler, 1998). The SRMR is a goodness of fit metric for PLS-SEM introduced by Henseler et al. (2014) that may be used to avoid model misspecification. The model is shown by values in Table 5 (0.068), which is less than the predefined threshold. So, the model is fitted.

**TABLE 5:** Model fit (standardized root means square residual).

	**Saturated model**	**Estimated model**
SRMR	0.062	0.068

R Square’s partial least square regression model shows us how much variation is explained by independent variables on the dependent variable and model strength. The goodness of fit of the model is shown in this analysis. It should have a value >0.3. The coefficient of determination (R square) values are >0.3, which is greater than the threshold value, indicating the goodness of the model in Table 6.

**TABLE 6:** R square—R^2^.

	**R square**	**R square adjusted**
Brand equity	0.695	0.691
Corporate reputation	0.851	0.850
Trust	0.710	0.708

Table 7 presents the value of CSR on CR as 3.161, which means acceptance of the H1 hypothesis because it forecasts that the CRS activities positively affect CR as this path that leads toward the CRS is significant statistically at 0.05 level. Thus, H1 confirms that socially responsible activities lead toward positive CR from the perspective of the consumer This finding also supports the previous studies on the subject where Nguyen and Nguyen (2017) stated that CSR positively correlates with CR. H2 suggested that CSR also has an affirmative effect on BE. The value of the direct impact of CSR on CR is 3.161, which is positive and supports H2. The findings are also aligned with the work of Marín et al. (2016). H3 suggested that CSR activities and initiatives directly affect the trust of consumers in brand image. Its value that ultimately CSR has an effect on trust also shows the positive relationship with CRS, which supports H3. The hypothesis is found to be significant at the 0.05 level. H4 claims that trust plays a mediating role between CSR and CR, while H5 claims that trust plays a partial mediating role between CSR and BE. Both have a positive effect and significant values, which means acceptance of H4 and H5, respectively.

**TABLE 7:** Path model results (direct and indirect).

	**Path**	**OS**	**Mean**	**S.D**	**T-values**	**P-values**
H1	CSR → Corporate reputation	0.470	0.438	0.149	3.161	0.002
H2	CSR → Brand equity	0.304	0.288	0.122	2.502	0.013
H3	CSR → Trust	0.843	0.845	0.040	21.309	0.000
H4	Trust → Brand equity	0.561	0.578	0.113	4.958	0.000
H5	Trust → Corporate reputation	0.492	0.524	0.146	3.370	0.001

**FIGURE 2:**
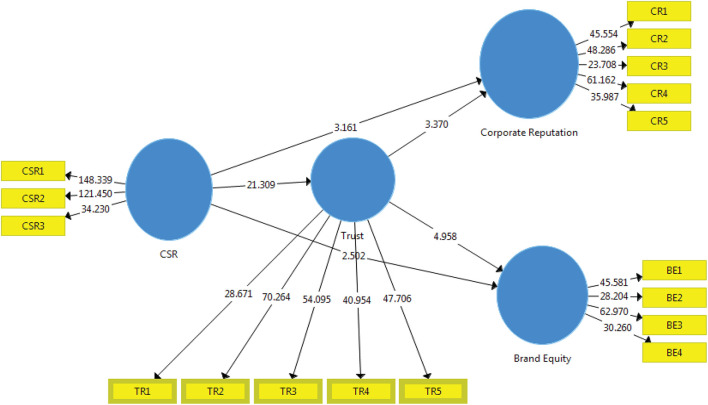
Theoretical constructs with R2 values.

Figure 2 depicts the relationship between all variables and mediation results. The inner model shows the relationship between the variables, whereas the outer model shows the factor loading values for each indicator. The connection between CSR and CR is 3.161, which means that the one-unit increase of CSR will positively impact CR by 3.161 points. By a 100% increase in CSR activities, it will increase CR by 316.1%. Similarly, CSR and BE connection values are 2.502, which also shows strong and positive relationships and that a one-unit increase in CSR initiatives will increase the BE of the company by 2.502 points. The mediating effect of trust shows that the mediating effect of Trust on CSR and CR as a consumer has a positive impact. Similarly, Figure 2 demonstrates the mediating impact of trust on the positive and robust relationship between CSR and BE.

## Discussion

The aim of this study was to investigate how CSR, directly and indirectly, influences CR and BE, using trust as a mediating variable. Previously, such studies were conducted in developed countries, such as the United States, United Kingdom, and other western countries. However, a limited amount of findings are argued and their findings are presented on emerging countries like Pakistan. So, the limited studies on the emerging countries have still shown the scarcity of findings from the banking sector perspective. Authors believe that financial institutions ought to identify their significant roles in the socio-economic development of any country. To the best of the knowledge of the author, this research is among the earliest research paradigms to investigate the impact of CSR on CR and BE in the Pakistani organizational context, especially by considering trust as a mediating variable. So, the exploration of this study is the role of trust as a mediating instrument. CSR activities help in the cumulative status of a corporate organization amongst its customers, employees, and stakeholders.

First, we focused on the direct relationship between CSR and CR and BE. The results show that CSR has a positive and significant impact on CR and BE, supporting our hypotheses H1 and H2. Prior studies have shown that CSR has a positive relationship with CR and BE (Hsu, 2012; Fatma et al., 2015; Lin and Chung, 2019; Lu et al., 2019). The finding of Hur et al. (2018) also support our results. They express in their study that CSR is known to be a standout amongst the best methods for promotion. There is just restricted research contemplating the impacts of CSR on advertising results. Therefore, this research indicates that CSR enhances CR and BE in the banking sector of Pakistan. Similarly, the RBV supports our study in the relationship between CRS, CR, and BE. Moreover, Rea et al. (2014) also suggest in their research that RBV supports the direct relationship among CSR, CR, and BE.

Second, this study depicts the positive and direct effects of CRS on trust, which supports Hypothesis 3. The finding of this study supports the research conducted by Choi and La (2013), which concludes that CSR significantly influences customer trust. Similarly, Park et al. (2014) examine the relationship between CSR and customer trust in Korean organizations, and the outcomes of their study confirmed that CSR is directly connected with customer trust. Thus, CRS has positively influenced customer trust in the banking sector of Pakistan. So, in case Pakistani banks want to create a positive image in the minds of their customers, they should focus on the CSR of the society that will affect the trust of the customer on their products.

Third, in this study, we test the mediated effect of trust between CSR, CR, and BE. It also translates significant results, which is a novel and original contribution in emerging countries like Pakistan, which supports H4 and H5. The mediated results support the findings of previous studies (Pivato et al., 2008; Fatma et al., 2015; Jalilvand et al., 2017). Forcadell and Aracil (2017) and Vlachos et al. (2009) inspected the mediating role of consumer satisfaction and trust among CSR and CR; however, they confirm in their study that trust is positively and significantly mediated between the CSR and CR. Further, Chang and Yeh (2017) proposed in their study that CSR has a positive relationship with BE using trust as a mediating variable. The outcomes of their study confirm that trust is intervening between CSR and brand equity. So, the outcomes of this research also indicated that customer trust as a critical variable positively affects CRS, CR, and BE. These results have significant inferences for PSBs in particular and private banks in Pakistan and advise that CSR activities may help in Constructing CR and BE.

## Conclusion

The results of this study confirmed the linkages among CSR, CR, and BE in the banking sector of Pakistan. Moreover, the finding of this research indicates that in the direct relationship, CSR positively influences CR and brand equity. The outcome of this study also confirms that trust positively and significantly mediates between CSR, CR, and brand equity. Furthermore, the outcomes confirmed that CSR has a positive and significant relationship with CR and brand equity.

This study highlight that the PSBs spend their resources on CSR activities to lead to significant advantages for partners and society. While participating in CSR exercises, PSBs will gain a progressively positive discernment and a reasonable frame of mind of their partners and stakeholders. Along with the fruitful insights, it is more important for banking and other industries to invest in the CSR interventions that can contribute to gain sustainable competitive advantage. Similarly, the investment in CSR is also fruitful for both stakeholders and the community. The exact outcomes of the examination show that CSR has an immediate positive and circuitous impact on CR and BE because findings here reveal that CSR indirectly influences CR as mediated by trust. Furthermore, discoveries uncover that the backhanded effects of CSR exercise on CR while mediated by trust. The multifaceted CSR demonstrated as a CR and BE are more viable explanations behind CSR exercises. Then again, buyer trust might be seen as a result of firms captivating CSR exercises. The solid connection between CSR activities and CR proves that an organization occupied with socially dependable exercises can anticipate different helpful results.

## Implications, Limitations, and Future Research Directions

### Managerial Implication

This research was conducted in the banking sector of Pakistan. The findings indicate some practical implications for the banking sector of Pakistan that could increase the organizational CR and BE through CSR. First, the managers need to create an environment of CSR activities. Those planning to do while establishing and maintaining strong relationships with firms and consumers through these CSR activities are more likely to create positive outcomes such as BE and CR (Javeed and Lefen, 2019). Second, managers, specifically in banking organizations should pay more attention to CSR activities and spend more of their resources on these activities. In this way, customers perceive the firm as more socially responsible and trustworthy than any other firm while considering it more favorably. This study shows that the results in a well-known or good reputation will increase brand equity. Third, the banking organizations and other investment-related companies also pay more close attention to their reputation in the minds of the consumers because it plays a critical role in evaluating the performance of the firm. Therefore, the banking organizations and investment-related firms should expect to enhance their brand while considering various socially responsible initiatives that positively impact society. The finding provides the most important insights to firm managers about their critical position in developing CSR as a customer brand partnership from their marketing viewpoints. They can take more effort to maintain deep trust between customers because it has been found that most consumers view a firm as more trustworthy when it is associated with some social problem.

### Limitation and Future Research Direction

This research has some limitations that may affect the generalization of its findings. First, due to time constraints and scared resources, the nature of our study was cross-sectional instead of a longitudinal design. Therefore, we recommend future researchers conduct a longitudinal mode study using CSR predictors on industrial BE and CR for more generalized results. Second, the data is collected from the second biggest city of Pakistan, an expensive city in Pakistan, affecting the buyers buying power. Such research will conduct in four to five different cities of the country, which will have generalized the study results. Third, the data was collected from the banking sector, which might cause common method bias. As the job nature of the target population is different from each other, future studies should focus on individuals who have similar jobs. In the future, such kind of study will explore the relationship between CSR and CR using the well-being of the customer as a mediating variable.

## Data Availability Statement

The original contributions presented in the study are included in the article/supplementary material, further inquiries can be directed to the corresponding authors.

## Author Contributions

MS and MA conceived the idea of the study. SR worked on the research methodology and helped in drafting the manuscript. MM worked on the results and analysis, and interpretation of model results. TO supervised the project and intensively edited the language of the manuscript. YZ approved and read the final manuscript and participated in the critical appraisal of the manuscript. All authors contributed to the article and approved the submitted version.

## Conflict of Interest

The authors declare that the research was conducted in the absence of any commercial or financial relationships that could be construed as a potential conflict of interest.

## Publisher’s Note

All claims expressed in this article are solely those of the authors and do not necessarily represent those of their affiliated organizations, or those of the publisher, the editors and the reviewers. Any product that may be evaluated in this article, or claim that may be made by its manufacturer, is not guaranteed or endorsed by the publisher.
